# Supplementary material for: G protein-coupled receptor-based thermosensation determines temperature acclimatization of Caenorhabditis elegans
Source: Nat Commun. 2024 Feb 23;15:1660. doi: 10.1038/s41467-024-46042-z (PMC10891075; doi:10.1038/s41467-024-46042-z)
Supplement: Supplementary file 1 — Supplementary Information [file 41467_2024_46042_MOESM1_ESM.pdf]

SUPPLEMENTARY INFORMATION

G protein-coupled receptor-based thermosensation determines temperature acclimatization of *Caenorhabditis elegans*

Kohei Ohnishi[1-3], Takaaki Sokabe[*4-7], Toru Miura[2,3], Makoto Tominaga[4-6], Akane Ohta[*1-3], Atsushi Kuhara[*1-3,7]

Supplementary Fig. 1 – Fig. 4

Supplementary Table 1 – Table 4

Supplementary Data 1. (separated excel file)

Supplementary Data 2. (separated excel file)

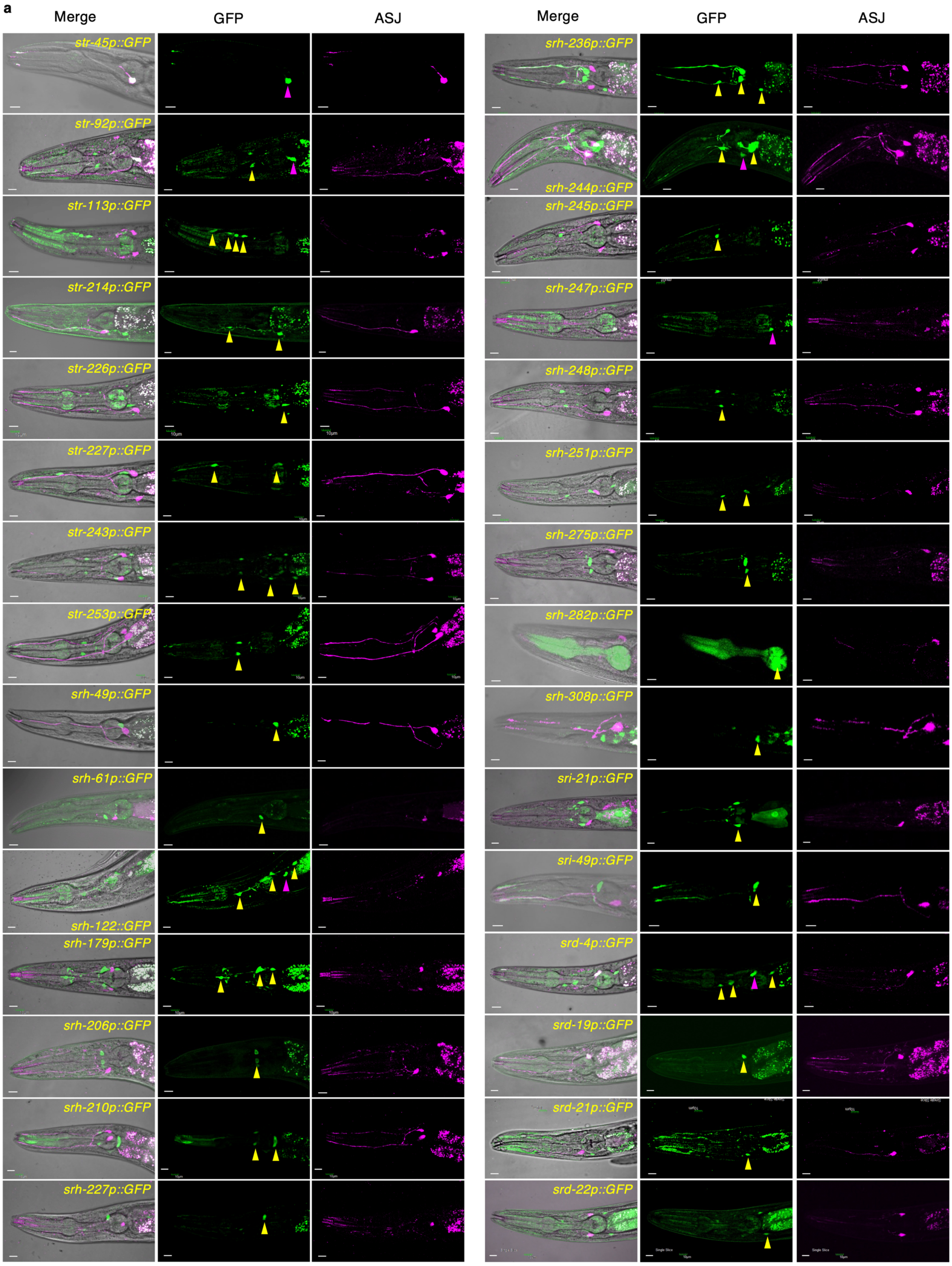
a
Merge
GFP
ASJ
Merge
GFP
ASJ
str-45p::GFP
str-92p::GFP
str-113p::GFP
str-214p::GFP
str-226p::GFP
str-227p::GFP
str-243p::GFP
str-253p::GFP
srh-49p::GFP
srh-61p::GFP
srh-122::GFP
srh-179p::GFP
srh-206p::GFP
srh-210p::GFP
srh-227p::GFP
srh-236p::GFP
srh-244p::GFP
srh-245p::GFP
srh-247p::GFP
srh-248p::GFP
srh-251p::GFP
srh-275p::GFP
srh-282p::GFP
srh-308p::GFP
sri-21p::GFP
sri-49p::GFP
srd-4p::GFP
srd-19p::GFP
srd-21p::GFP
srd-22p::GFP

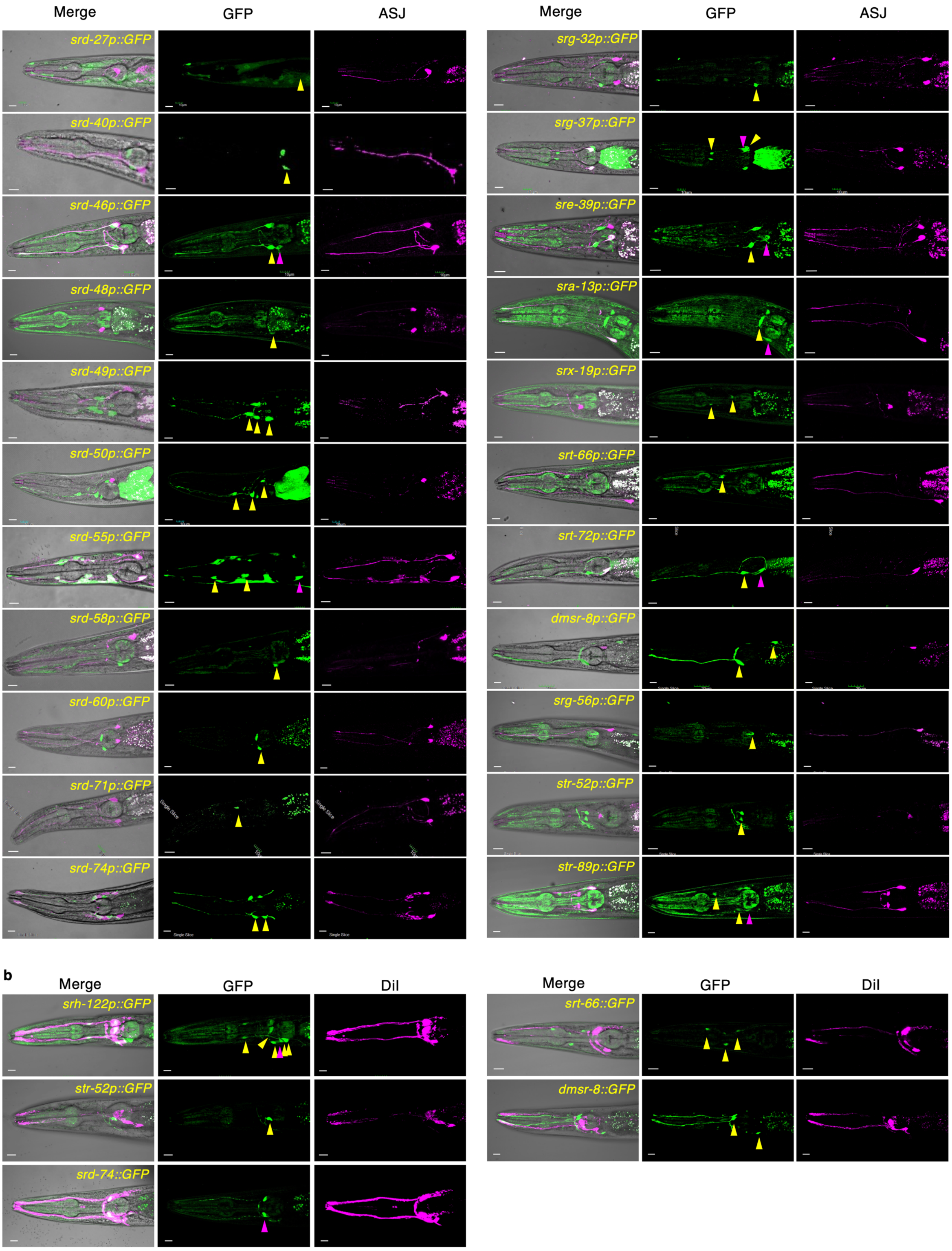

Supplementary Fig. 1. Expression patterns of 50 GPCR genes. Expression patterns visualized in transgenic animals, wherein GFP was expressed under the upstream promoter regions of each GPCR gene (Green). **a**, GFP fluorescence was merged with *trx-1p::DsRedm* that labels ASJ (Magenta). **b**, GFP fluorescence were merged with DiI that labels six pairs of amphid neurons in the head with red fluorescence (Magenta). All scale bars indicate 10 µm. Magenta or yellow arrowheads indicate GFP fluorescence in the cell body of ASJ or ADL thermosensory neuron, respectively.

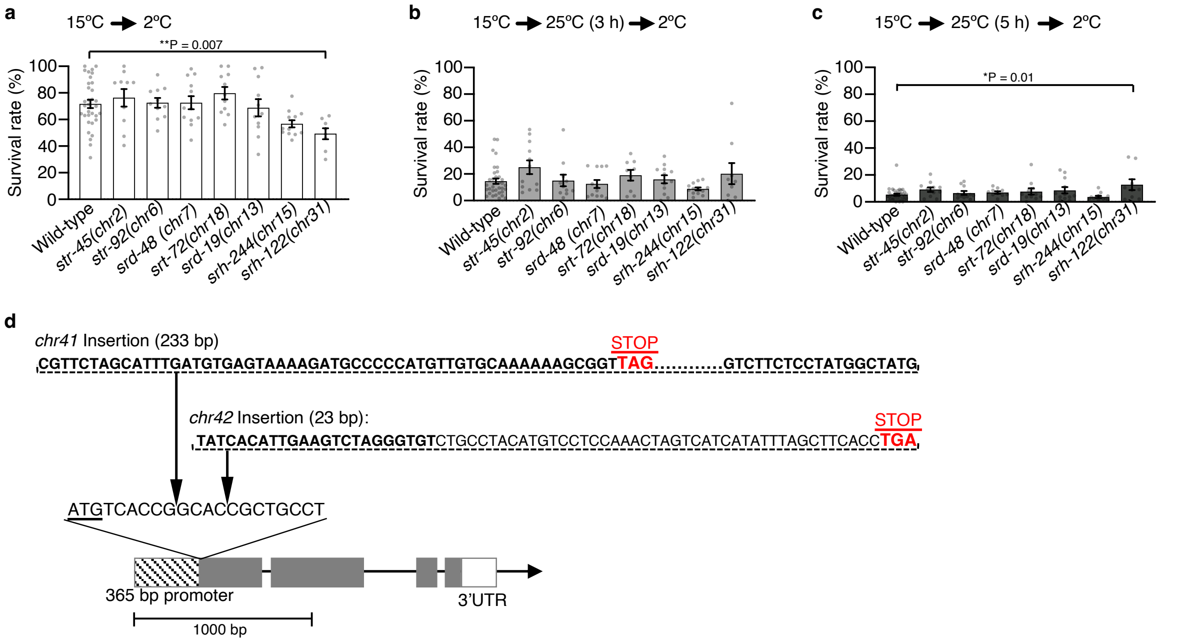

Supplementary Fig. 2. Temperature acclimatization of knockout mutants of 12 GPCRs. a–c, Temperature acclimatization of GPCR knockout mutants constructed by CRISPR/Cas9 and assayed under [15°C→25°C (0, 3, or 5 h)→2°C]. n = 33, 10, 11, 12, 11, 11, 12, and 8 (a), n = 34, 12, 11, 12, 9, 12, 13, and 9 (b), n = 35, 11, 11, 10, 11, 12, 13, and 9 (c). n indicates independent experiments (shown from left in the bar graph). Bar graphs represent mean ± SEM. p-values were calculated using two-sided one-way ANOVA with Dunnett's test (a–f). n.s. $P \geq 0.05$; $^{*}P < 0.05$; $^{**}P < 0.01$. Source data are provided as a Source Data file. **d**, Genomic structure of *srh-40* and the KO mutants. The shaded box and gray boxes indicate the promotor region and exons, respectively. The positions of nonsense mutations caused by each insertion in *chr41* and *chr42* are shown. In each mutant, an aberrant stop codon occurred in the first exon by frameshift.

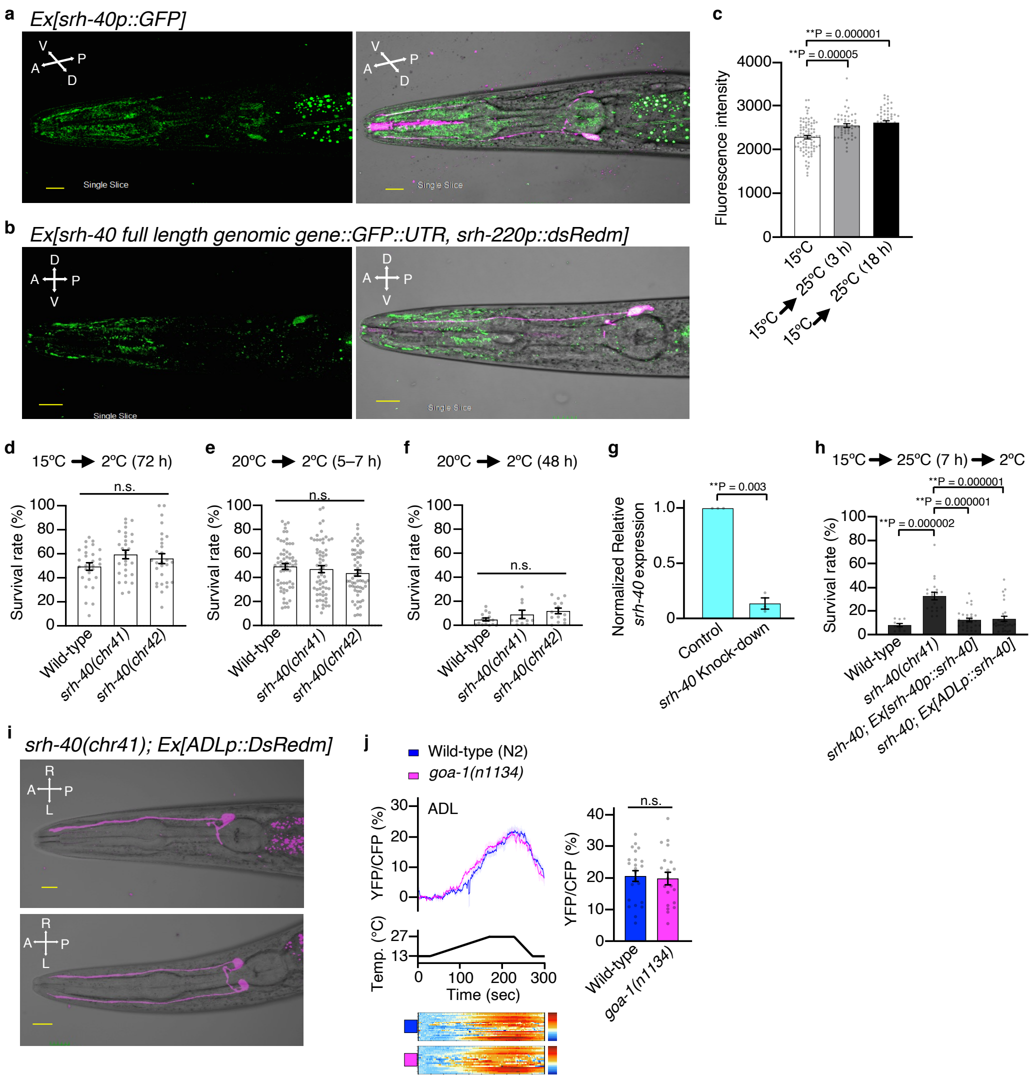

Supplementary Fig. 3. Expression analysis, cold tolerance, and Ca^{2+} imaging. a, **b**, Wild-type expressing *srh-40p::gfp* (a) or *srh-40 full length::gfp* (b), and *srh-220p::DsRedm* that labels ADL (Magenta). **c**, GFP fluorescence intensity induced by the *srh-40* promoter in the ADL of wild-type grown at 15°C, 15°C→25°C (3 h), or 15°C→25°C (18 h) (n = 88, 49, and 57). **d**–**f** Cold tolerance of *srh-40* mutants ([15°C→2°C (72 h)], n = 24) or [20°C→2°C (5–7 h); n = 60, 60, and 59) to observe the mutant phenotype under conditions where the survival rate of the wild-type is approximately 50%, and ([20°C→2°C (48 h)]; n = 18, 13, and 17). **g**, Relative *srh-40* mRNA expression in *eri-1; lin-15B* mutant in which knocked-down *srh-40* gene, measured by qPCR. Bars represent fold changes relative to control with no-treated *eri-1; lin-15B* mutant. n = 3. **h**, Temperature acclimatization of the rescued *srh-40* mutant ([15°C→25°C (7 h)→2°C], n = 10, 20, 30, 30). **i**, Representative confocal image of ADL in the *srh-40* mutant. n = 39. A, anterior; P, posterior; D, dorsal; V, ventral; L, left; R, right. **j**, Ca^{2+} imaging of ADL in *goa-1* mutant (n = 23, 20). Traces indicate the averaged YFP/CFP ratio of YC3.60 in response to warming and cooling. Bar graphs indicate the averaged YFP/CFP ratio between 211–220 sec, the maximum point in each strain. Each row in the color maps represents relative changes in Ca^{2+} concentrations from one worm; excluded values >100% are shown in white. The colors of bar graphs and color maps correspond to the colors of response traces. Scale bars indicate 10 µm (a, b, i). n indicates independent experiments (shown from left in the bar graph) (c–h, j). Bar graphs represent mean ± SEM. p-values were calculated using unpaired *t*-test (g, j), one-way ANOVA with Dunnett's test (d–f) or Tukey–Kramer's test (c), and Kruskal–Wallis's test with Steel–Dwass's test (h). n.s. $P \geq 0.05$; $^{**}P < 0.01$. All statistical tests were two-sided. Source data are provided as a Source Data file.

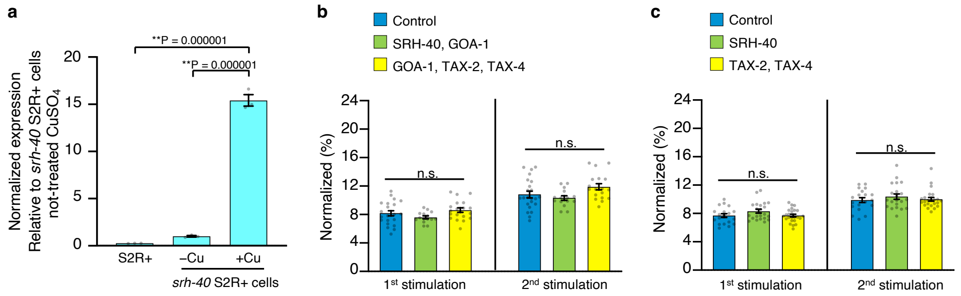

Fig 4. Ca^{2+} imaging of *Drosophila* S2R+ cells for negative control. a, Relative *srh-40* mRNA expression in naïve S2R+ cells, or *srh-40* stable S2R+ cells treated or not treated with $CuSO_4$, measured by qPCR. Bars represent fold changes relative to *srh-40* stable S2R+ cells that were not treated with $CuSO_4$. n = 3. **b**, **c**, Comparison of the maximum increase in $Ca^{2+}{}_i$ concentrations in response to the first and second heat stimulation in the control and cells expressing various genes as indicated. n= 828, 538, and 688 (951, 538 and 652 in second stimuli) cells examined over 21, 15, and 19 (23, 15, and 18 in second stimuli) independent experiments (b), n= 630, 758, and 766 (630, 731, and 728 in second stimuli) cells examined over 19, 22, and 24 (19, 21, and 23 in second stimuli) independent experiments (c). n indicates independent experiments (shown from left in the bar graph). Bar graphs represent mean ± SEM. p-values were calculated using one-way ANOVA with Dunnett's test (c and first stimuli in b) or Tukey–Kramer's test (a), and Kruskal–Wallis's test with Steel's test (second stimuli in b). n.s. $P \geq 0.05$; $^{**}P < 0.01$. All statistical tests were two-sided. Source data are provided as a Source Data file.

Supplementary Table 1. GPCRs expressed in thermosensory neurons.

Gene	Sequence name	ASJ	ADL
str-43	T23D5.9	ND (CeNGEN −)	ND (CeNGEN −)
str-45	T09F5.3	+	–
str-52	C45H4.12	–	+
str-89	F59A1.4	+	ND (CeNGEN −)
str-92	F59A1.3	+	–
str-113	Y39H10B.1	–	–
str-214	C31A11.9	–	–
str-226	C07G3.4	–	ND (CeNGEN NO DATA)
str-227	C07G3.3	–	ND (CeNGEN −)
str-243	K02H11.7	–	ND (CeNGEN −)
str-253	F41B5.8	–	–
sra-13	F49E12.5	+	ND (CeNGEN -)
srh-40	Y40D12A.3	–	+
srh-49	C10G11.4	– (CeNGEN +)	ND (CeNGEN −)
srh-61	T21B4.8	–	ND (CeNGEN −)
srh-122	R08H2.5	+	ND (CeNGEN NO DATA)
srh-179	ZK228.7	–	ND (CeNGEN −)
srh-206	Y102A5C.15	–	ND (CeNGEN +)
srh-210	D1065.4	– (CeNGEN +)	ND (CeNGEN +)
srh-227	C35D6.2	–	ND (CeNGEN NO DATA)
srh-236	Y68A4A.9	–	ND (CeNGEN NO DATA)
srh-244	C03G6.9	+	ND (CeNGEN −)
srh-282	T21D12.5	– (CeNGEN +)	–
srh-308	F58E10.6	–	+
sri-21	T24A6.4	–	ND (CeNGEN −)
sri-49	F22E5.14	–	ND (CeNGEN −)
srd-4	ZK863.5	+	ND (CeNGEN −)
srd-19	F53F1.11	+	– (CeNGEN +)
srd-21	W06G6.3	–	–
srd-22	F26G5.3	–	–
srd-27	T26E4.12	–	–
srd-40	F17A2.12	–	ND (CeNGEN −)
srd-46	F17A2.10	+	ND (CeNGEN −)
srd-48	F17A2.9	– (CeNGEN +)	–
srd-49	R04B5.8	–	ND (CeNGEN −)
srd-50	F15A2.4	–	ND (CeNGEN −)
srd-55	K02A2.2	+	ND (CeNGEN −)
srd-58	E04F6.13	–	ND (CeNGEN NO DATA)
srd-60	C13B7.3	–	ND (CeNGEN NO DATA)
srd-71	C13B7.4	–	ND (CeNGEN NO DATA)
srd-74	C24H11.4	–	–
sre-39	F10G7.7	+	ND (CeNGEN +)
srg-32	T21C9.7	–	ND (CeNGEN -)
srg-37	K04C1.1	+	–

srh-245	F31F4.9	–	–
srh-247	C31B8.13	+	– (CeNGEN NO DATA)
srh-248	H05B21.2	–	ND (CeNGEN NO DATA)
srh-251	R08H2.4	–	ND (CeNGEN NO DATA)
srh-275	C03G6.7	– (CeNGEN +)	ND (CeNGEN +)

srg-56	C24B9.10	–	ND (CeNGEN +)
srt-66	B0238.14	–	–
srt-72	C36C5.9	+	ND (CeNGEN −)
srx-19	T05A12.1	–	ND (CeNGEN −)
dmsr-8	C35A5.7	–	ND (CeNGEN −)

"+" and "–" indicate positive and negative GFP expression driven by each gene promoter in this study, respectively. ND indicates "not determined"; "(CeNGEN +)" and "(CeNGEN −)" indicate positive and negative expression in CeNGEN, *the C. elegans* Neuronal Gene Expression database (Select threshold: 2), respectively.

Supplementary Table 2. *C. elegans* strain

Genotype	Strains	Source
eri-1(mg366); lin-15B(n744)	KP3948	CGC
tax-4(p678)	PR678	CGC
osm-9(ky10)	CX10	CGC
ocr-2(ak47)	CX4544	CGC
osm-9(ky10); ocr-2(ak47)	CX4652	CGC
osm-9(ky10) ocr-2(ak47); ocr-1(ak46)	FG125	CGC
srh-40(chr41); osm-9(ky10) ocr-2(ak47); ocr-1(ak46)	–	This study
srh-40(chr41); osm-9(ky10) ocr-2(ak47)	–	This study
egl-30(ad806)	DA1084	CGC
egl-30(ep271)	CE1047	CGC
gcy-5(tm897)	OH4844	CGC
glr-3(tm6403)	–	NBRP
Ex[flp-6p::CeGCaMP8, gcy-5p::tagRFP]	–	Takagaki et al., 2019
glr-3(tm6403); Ex[flp-6p::CeGCaMP8, gcy-5p::tagRFP]	–	Ohnishi et al. 2020
Ex[ges-1p::NLS::GFP, AIYp::GFP, pBluescript Ⅱ SK+]	–	Ujisawa et al., 2016
Ex[sre-1p::yc3.60, rol-6(gf)]	–	Ujisawa et al., 2016
gpa-3(pk35)	NL335	CGC
goa-1(n1134)	MT2426	CGC
goa-1(n1134); gpa-3(pk35)	KHR80	Ujisawa et al., 2016
ExUDA30[str-45 full length genomic gene::gfp, trx-1p::DsRedm, rol-6(gf)]	–	This study
Ex[str-92p::gfp, trx-1p::NLS::DsRedm, rol-6(gf)]	–	This study
Ex[str-113p::gfp, trx-1p::DsRedm, rol-6(gf)]	–	This study
Ex[str-214p::gfp, trx-1p::DsRedm, rol-6(gf)]	–	This study
Ex[str-226p::gfp, trx-1p::DsRedm, rol-6(gf)]	–	This study
Ex[str-227p::gfp, trx-1p::DsRedm, rol-6(gf)]	–	This study
Ex[str-243p::gfp, trx-1p::DsRedm, rol-6(gf)]	–	This study
Ex[str-253p::gfp, trx-1p::DsRedm, rol-6(gf)]	–	This study
Ex[srh-49p::gfp, trx-1p::DsRedm, rol-6(gf)]	–	This study
ExUDA32[srh-61p::gfp, trx-1p::DsRedm, rol-6(gf)]	–	This study
Ex[srh-122p::gfp, trx-1p::DsRedm, rol-6(gf)]	–	This study
Ex[srh-179p::gfp, trx-1p::DsRedm, rol-6(gf)]	–	This study
Ex[srh-206p::gfp, trx-1p::DsRedm, rol-6(gf)]	–	This study
Ex[srh-210p::gfp, trx-1p::DsRedm, rol-6(gf)]	–	This study
Ex[srh-227p::gfp, trx-1p::DsRedm, rol-6(gf)]	–	This study
Ex[srh-236p::gfp, trx-1p::DsRedm, rol-6(gf)]	–	This study
Ex[srh-245p::gfp, trx-1p::DsRedm, rol-6(gf)]	–	This study
Ex[srh-247p::gfp, trx-1p::DsRedm, rol-6(gf)]	–	This study
Ex[srh-248p::gfp, trx-1p::DsRedm, rol-6(gf)]	–	This study
Ex[srh-251p::gfp, trx-1p::DsRedm, rol-6(gf)]	–	This study
Ex[srh-275p::gfp, trx-1p::DsRedm, rol-6(gf)]	–	This study
ExUDA34[srh-282p::gfp, trx-1p::DsRedm, rol-6(gf)]	–	This study
ExUDA28[srh-308p::gfp, trx-1p::DsRedm, rol-6(gf)]	–	This study

Ex[sri-21p::gfp, trx-1p::DsRedm, rol-6(gf)]	–	This study
Ex[srd-4p::gfp, trx-1p::DsRedm, rol-6(gf)]	–	This study
ExUDA27[sri-49p::gfp, trx-1p::DsRedm, rol-6(gf)]	–	This study
ExUDA45[srd-19p::gfp, trx-1p::DsRedm, rol-6(gf)]	–	This study
Ex[srd-21p::gfp, trx-1p::DsRedm, rol-6(gf)]	–	This study
Ex[srd-22p::gfp, trx-1p::DsRedm, rol-6(gf)]	–	This study
Ex[srd-27p::gfp, trx-1p::DsRedm, rol-6(gf)]	–	This study
ExUDA42[srd-40p::gfp, trx-1p::DsRedm, rol-6(gf)]	–	This study
ExUDA39[srd-46p::gfp, trx-1p::DsRedm, rol-6(gf)]	–	This study
ExUDA40[srd-48p::gfp, trx-1p::DsRedm, rol-6(gf)]	–	This study
ExUDA48[srd-49p::gfp, trx-1p::DsRedm, rol-6(gf)]	–	This study
Ex[srd-50p::gfp, trx-1p::DsRedm, rol-6(gf)]	–	This study
Ex[srd-55p::gfp, trx-1p::DsRedm, rol-6(gf)]	–	This study
Ex[srd-58p::gfp, trx-1p::DsRedm, rol-6(gf)]	–	This study
Ex[srd-60p::gfp, trx-1p::DsRedm, rol-6(gf)]	–	This study
Ex[srd-71p::gfp, trx-1p::DsRedm, rol-6(gf)]	–	This study
Ex[srd-74p::gfp, trx-1p::DsRedm, rol-6(gf)]	–	This study
Ex[srg-32p::gfp, trx-1p::DsRedm, rol-6(gf)]	–	This study
Ex[srg-37p::gfp, trx-1p::DsRedm, rol-6(gf)]	–	This study
Ex[sre-39p::gfp, trx-1p::DsRedm, rol-6(gf)]	–	This study
ExUDA29[srx-19p::gfp, trx-1p::DsRedm, rol-6(gf)]	–	This study
Ex[sra-13p::gfp, trx-1p::DsRedm, rol-6(gf)]	–	This study
Ex[srt-66p::gfp, trx-1p::DsRedm, rol-6(gf)]	–	This study
Ex[srt-72p::gfp, trx-1p::DsRedm, rol-6(gf)]	–	This study
Ex[dmsr-8p::gfp, trx-1p::DsRedm, rol-6(gf)]	–	This study
Ex[srh-40p::gfp, trx-1p::DsRedm, rol-6(gf)]	–	This study
Ex[srh-40 full length genomic gene::gfp::srh-40 3'UTR, trx-1p::DsRedm, rol-6(gf)]	–	This study
Ex[srg-56p::gfp, trx-1p::DsRedm, rol-6(gf)]	–	This study
Ex[str-52p::gfp, trx-1p::DsRedm, rol-6(gf)]	–	This study
Ex[str-89p::gfp, trx-1p::DsRedm, rol-6(gf)]	–	This study
str-45(chr2)	KHR44	This study
str-45(chr3)	KHR45	This study
str-92(chr4)	KHR61	This study
str-92(chr6)	KHR63	This study
srd-48(chr7)	KHR70	This study
srd-48(chr8)	KHR71	This study
srd-19(chr13)	KHR98	This study
srd-19(chr14)	KHR99	This study
srh-244(chr15)	KHR100	This study
srh-244(chr16)	KHR102	This study
srt-72(chr18)	KHR090	This study
srh-122(chr31)	KHR123	This study
str-43(chr39)	KHR151	This study
srg-56(chr40)	KHR152	This study
srh-40(chr41)	KHR153	This study
srh-40(chr42)	KHR154	This study
str-52(chr43)	KHR155	This study

str-89(chr44)	KHR156	This study
str-89(chr45)	KHR157	This study
glr-3(tm6403) backcross 3 times	KHR197	This study
Ex[srh-220p::DsRedm, rol-6(gf)]	–	This study
srh-40(chr42); Ex[ges-1p::NLS::gfp, AIYp::gfp, pBluescript Ⅱ SK+]	–	This study
srh-40(chr42); Ex[srh-40p::srh-40 cDNA, ges-1p::NLS::gfp, AIYp::gfp, pBluescript Ⅱ SK+]	–	This study
srh-40(chr42); Ex[srh-220p::srh-40 cDNA, ges-1p::NLS::gfp, AIYp:gfp, pBluescript Ⅱ SK+]	–	This study
srh-40(chr42); Ex[sre-1p::yc3.60, rol-6(gf)]	–	This study
srh-40(chr42); Ex[srh-220p::srh-40cDNA, sre-1p::yc3.60, rol-6(gf)]	–	This study
Ex[srh-220p::egl-30sense, srh-220p::egl-30anti-sense, AIYp::GFP, ges-1p::NLS GFP, pBluescript Ⅱ SK+]	–	This study
Ex[srh-220p::egl-30(Q205L) cDNA, AIYp::gfp, ges-1p::NLS::gfp, pBluescript Ⅱ SK+]	–	This study
Ex[srh-220p::egl-30sense, srh-220p::egl-30anti-sense, sre-1p::yc3.60, rol-6(gf)]	–	This study
Ex[srh-220p::egl-30(Q205L) cDNA, sre-1p::yc3.60, rol-6(gf)]	–	This study
Ex[srh-220p::srh-40, sre-1p::yc3.60, rol-6(gf)]	–	This study
Ex[srh-220p::srh-40, sre-1p::yc3.60, srh-220p::egl-30sense, srh-220p::egl-30anti-sense, sre-1p::yc3.60, rol-6(gf)]	–	This study
Ex[sre-1p::yc4.12, rol-6(gf)]	–	This study
ocr-2(ak47); Ex[sre-1p::yc3.60, rol-6(gf)]	–	Ohnishi et al. 2020
srh-40(chr41); ocr-2(ak47); Ex[srh-220p::srh-40, sre-1p::yc3.60, rol-6(gf)]	–	This study
srh-40(chr41); osm-9(ky10) ocr-2(ak47); ocr-1(ak46); Ex[sre-1p::yc3.60, rol-6(gf)]	–	This study
srh-40(chr41); osm-9(ky10) ocr-2(ak47); Ex[sre-1p::yc3.60, rol-6(gf)]	–	This study
goa-1(n1134); Ex[sre-1p::yc3.60, rol-6(gf)]	–	This study
Ex[flp-6p::CeG-CaMP8, gcy-5p::tagRFP, gcy-5p::srh-40]	–	This study
glr-3(tm6403); Ex[flp-6p::CeG-CaMP8, gcy-5p::tagRFP, gcy-5p::srh-40]	–	This study
goa-1(n1134); gpa-3(pk35); Ex[flp-6p::CeG-CaMP8, gcy-5p::tagRFP, gcy-5p::srh-40]	–	This study
gpa-3(pk35); Ex[flp-6p::CeG-CaMP8, gcy-5p::tagRFP, gcy-5p::srh-40]	–	This study
goa-1(n1134); Ex[flp-6p::CeG-CaMP8, gcy-5p::tagRFP, gcy-5p::srh-40]	–	This study
goa-1(n1134); gpa-3(pk35); Ex[flp-6p::CeG-CaMP8, gcy-5p::tagRFP]	–	This study
gpa-3(pk35); Ex[flp-6p::CeG-CaMP8, gcy-5p::tagRFP]	–	This study
goa-1(n1134); Ex[flp-6p::CeG-CaMP8, gcy-5p::tagRFP]	–	This study
gcy-5(tm897); Ex[flp-6p::CeG-CaMP8, gcy-5p::tagRFP, gcy-5p::srh-40]	–	This study
gcy-5(tm897); Ex[flp-6p::CeG-CaMP8, gcy-5p::tagRFP]	–	This study
tax-4(p678); Ex[flp-6p::CeG-CaMP8, gcy-5p::tagRFP, gcy-5p::srh-40]	–	This study
tax-4(p678); Ex[flp-6p::CeG-CaMP8, gcy-5p::tagRFP]	–	This study
egl-30(ad806); Ex[flp-6p::CeG-CaMP8, gcy-5p::tagRFP]	–	This study
egl-30(ad806); Ex[flp-6p::CeG-CaMP8, gcy-5p::tagRFP, gcy-5p::srh-40]	–	This study

CGC: The *Caenorhabditis* Genetic Center

NBRP: The National BioResource Project (Japan)

Supplementary Table 3. The mutation of mutants constructing by CRISPR/Cas9.

Strain	Allele	Gene	Mutation
KHR44	*chr2*	*str-45*	Insertion of 5 bp containing stop codon and NheI restriction site in 1st exon
KHR45	*chr3*	*str-45*	Deletion of 243 bp in 1st exon.
KHR61	*chr4*	*str-92*	Deletion of 527 bp and insertion of 3 bp in 3rd and 4th exon.
KHR62	*chr5*	*str-92*	Deletion of 626 bp and insertion of 96 bp in 3rd, 4th and 5th exon.
KHR70	*chr7*	*srd-48*	Insertion of 4 bp NheI restriction site containing stop codon in 3rd exon.
KHR71	*chr8*	*srd-48*	Insertion 16 bp and stop codon (TGA) in 1st exon.
KHR98	*chr13*	*srd-19*	Deletion of 502 bp in 2nd and 3rd exon.
KHR99	*chr14*	*srd-19*	Deletion of 299 bp in 2nd and 3rd exon.
KHR100	*chr15*	*srh-244*	Insertion of 5 bp containing stop codon and NheI restriction site in 2nd exon.
KHR102	*chr16*	*srh-244*	Deletion of about 400 bp in 2nd and 3rd exon.
KHR090	*chr18*	*srt-72*	Insertion of 3 bp, which induce non-sense mutation.
KHR123	*chr31*	*srh-122*	Deletion of 4 bp and insertion 26 bp in 1st exon.
KHR151	*chr39*	*str-43*	Insertion of 93 bp containing stop codon in 1st exon.
KHR152	*chr40*	*srg-56*	Insertion of 2 bp in 1st exon, which induce non-sense mutation.
KHR153	*chr41*	*srh-40*	Insertion of 233 bp in 1st exon, which induce non-sense mutation.
KHR154	*chr42*	*srh-40*	Insertion of 23 bp in 1st exon, which induce frame shift and non-sense mutation.
KHR155	*chr43*	*str-52*	Insertion 74 bp in 1st exon, which induce non-sense mutation.
KHR156	*chr44*	*str-89*	Insertion of 113 bp in 1st exon, which induce non-sense mutation.
KHR157	*chr45*	*str-89*	Deletion of 134 bp containing start codon in 1st exon.

Supplementary Table 4. The plasmids in this paper

Name	Construct
**Expression analysis**	
pMAI1	*str-45 full length genomic gene::gfp*
pMAI3	*srh-308p::gfp*
pMAI4	*sri-49p::gfp*
pMAI5	*srx-19p::gfp*
pUDA23	*str-214p::gfp*
pUDA24	*srh-282p::gfp*
pUDA27	*srd-46p::gfp*
pUDA28	*srd-48p::gfp*
pUDA30	*srd-40p::gfp*
pUDA31	*srd-50p::gfp*
pUDA32	*srh-61p::gfp*
pUDA37	*srd-19p::gfp*
pUDA39	*srd-49p::gfp*
pMIU001	*srd-71p::gfp*
pMIU002	*srd-74p::gfp*
pMIU003	*str-113p::gfp*
pMIU004	*srd-21p::gfp*
pMIU005	*srd-22p::gfp*
pMIU006	*srh-122p_gfp*
pMIU007	*sra-13p::gfp*
pMIU008	*srt-66p::gfp*
pMIU009	*srt-72p::gfp*
pMIU010	*dmsr-8p::gfp*
pKOH003	*srd-4p::gfp*
pKOH004	*srg-37p::gfp*
pKOH011	*str-92p::gfp*
pKOH012	*str-227p::gfp*
pKOH013	*str-253p::gfp*
pKOH014	*str-243p::gfp*
pKOH015	*srh-49p::gfp*
pKOH023	*srd-55p::gfp*
pKOH024	*srd-60p::gfp*
pKOH026	*str-226p::gfp*
pKOH027	*srd-27p::gfp*
pKOH029	*srh-179p::gfp*

Name	Construct
**For CRISPR/Cas9 system**	
pKOH020	*str-45 gRNA_no.5*
pKOH010	*dpy-10 gRNA*
pKOH021	*str-92 gRNA no.2*
pKOH022	*str-92 gRNA no.3*
pKOH019	*srd-48 gRNA*
pKOH034	*srd-19 gRNA no.1*
pKOH055	*srd-19 gRNA no.2*
pKOH046	*srh-208 gRNA*
pKOH047	*srh-244 gRNA*
pMIU014	*srt-72 gRNA*
pMIU015	*srh-122 gRNA no.1*
pMIU016	*srh-122 gRNA no.2*
pMIU017	*srh-40 gRNA*
pMIU018	*srg-56 gRNA*
pMIU019	*str-43 gRNA*
pMIU020	*str-52 gRNA*
pMIU021	*str-89 gRNA*
**For analysis of *srh-40* in *C. elegans*.**	
pKOH141	*srh-220p::DsRedm*
pMIU022	*srh-40p::gfp*
pMIU066	*srh-40 full length genomic gene::gfp:: srh-40 3'UTR*
pMIU028	*srh-220p::srh-40cDNA*
pMIU029	*srh-40p::srh-40cDNA*
pMIU030	*gcy-5p::srh-40cDNA*
pKOH241	*pPD95.75-egl30(Q205L)*
pKOH244	*srh-220p::egl-30(Q205L)cDNA*
pKOH252	*srh-220p::egl-30 anti-sense*
pKOH253	*srh-220p::egl-30 sense*
pMIU156	*sre-1p::yc4.12*
**Ca^{2+} imaging in S2R+ cells.**	
pMIU097	*pMT srh-40*
pMIU136	*pAc5.1 osm-9::T2A::ocr-2*
pMIU133	*pMT egl-30::T2A::mCherry*
pKOH297	*pAc5.1 tax-2::T2A::tax-4*
pMIU146	*pAc5.1 goa-1::T2A::mCherry*

pKOH030	*srh-206p::gfp*
pKOH032	*srh-210p::gfp*
pKOH035	*srh-227p::gfp*
pKOH036	*srh-236p::gfp*
pKOH037	*srh-244p::gfp*
pKOH038	*srh-245p::gfp*
pKOH039	*srh-247p::gfp*
pKOH040	*srh-248p::gfp*
pKOH041	*srh-251p::gfp*
pKOH042	*srh-275p::gfp*
pKOH043	*sre-39p::gfp*
pKOH044	*sri-21p::gfp*
pKOH045	*srg-32p::gfp*
pMIU023	*srg-56p::gfp*
pMIU024	*str-52p::gfp*
pMIU025	*str-89p::gfp*

pKOH233	*pAc5.1-mCherry*
