# Supplementary material for: G protein-coupled receptor-based thermosensation determines temperature acclimatization of Caenorhabditis elegans
Source: Nat Commun. 2024 Feb 23;15:1660. doi: 10.1038/s41467-024-46042-z (PMC10891075; doi:10.1038/s41467-024-46042-z)
Supplement: Supplementary file 3 — Description of Additional Supplementary Files [file 41467_2024_46042_MOESM3_ESM.pdf]

# DESCRIPTION OF ADDITIOANAL SUPPLEMENTARY FILES DOCUMENT

**Supplementary Data 1:** Raw data of cold tolerance in GPCR knockdown animals.

**Supplementary Data 2:** Sequences of oligonucleotides.
