# Supplementary material for: G protein-coupled receptor-based thermosensation determines temperature acclimatization of Caenorhabditis elegans
Source: Nat Commun. 2024 Feb 23;15:1660. doi: 10.1038/s41467-024-46042-z (PMC10891075; doi:10.1038/s41467-024-46042-z)
Supplement: Supplementary file 6 — Reporting Summary [file 41467_2024_46042_MOESM6_ESM.pdf]

nature portfolio

Corresponding author(s): Atsushi Kuhara

Last updated by author(s): 2024/1/8

Reporting Summary

Nature Portfolio wishes to improve the reproducibility of the work that we publish. This form provides structure for consistency and transparency in reporting. For further information on Nature Portfolio policies, see our Editorial Policies and the Editorial Policy Checklist.

Statistics

For all statistical analyses, confirm that the following items are present in the figure legend, table legend, main text, or Methods section.

n/a	Confirmed	
☐	☒	The exact sample size (*n*) for each experimental group/condition, given as a discrete number and unit of measurement
☐	☒	A statement on whether measurements were taken from distinct samples or whether the same sample was measured repeatedly
☐	☒	The statistical test(s) used AND whether they are one- or two-sided<br>*Only common tests should be described solely by name; describe more complex techniques in the Methods section.*
☐	☒	A description of all covariates tested
☐	☒	A description of any assumptions or corrections, such as tests of normality and adjustment for multiple comparisons
☐	☒	A full description of the statistical parameters including central tendency (e.g. means) or other basic estimates (e.g. regression coefficient) AND variation (e.g. standard deviation) or associated estimates of uncertainty (e.g. confidence intervals)
☐	☒	For null hypothesis testing, the test statistic (e.g. F, t, r) with confidence intervals, effect sizes, degrees of freedom and P value noted<br>*Give P values as exact values whenever suitable.*
☒	☐	For Bayesian analysis, information on the choice of priors and Markov chain Monte Carlo settings
☒	☐	For hierarchical and complex designs, identification of the appropriate level for tests and full reporting of outcomes
☒	☐	Estimates of effect sizes (e.g. Cohen's *d*, Pearson's *r*), indicating how they were calculated

Our web collection on statistics for biologists contains articles on many of the points above.

Software and code

Policy information about availability of computer code

Data collection	In calcium imaging of C. elegans, fluorescence intensities were acquired using MetaMorph ver.7.10 (Molecular Devices) image analysis software. In calcium imaging of S2R+ cells, Fura-2 ratiometric intensities were acquired using NIS-Elements Advanced Research ver.5.30.05 (Nikon) imaging software. Fluorescence images by confocal laser microscopy were acquired using the FV10-ASW (Ver. 04.02) software (Olympus).
Data analysis	In statistical analysis, we used "MAC statistical analysis version 3(ESUMI)" software on Microsoft EXCEL. Dots representing individual data shown in all bar charts were created by GraphPad Prism9. All heatmaps for calcium imaging analysis were created in GraphPad Prism 9.<br>In calcium imaging of C. elegans, fluorescence intensities were processed using MetaMorph ver.7.10 (Molecular Devices) image analysis software. In calcium imaging of S2R+ cells, Fura-2 ratiometric intensities were processed using NIS-Elements Advanced Research ver.5.30.05 (Nikon) imaging software.

For manuscripts utilizing custom algorithms or software that are central to the research but not yet described in published literature, software must be made available to editors and reviewers. We strongly encourage code deposition in a community repository (e.g. GitHub). See the Nature Portfolio guidelines for submitting code & software for further information.

Data

Policy information about availability of data

All manuscripts must include a data availability statement. This statement should provide the following information, where applicable:

- Accession codes, unique identifiers, or web links for publicly available datasets
- A description of any restrictions on data availability
- For clinical datasets or third party data, please ensure that the statement adheres to our policy

The data generated during in this study are available within this article and its supplementary information or from the corresponding author on reasonable request. Source data are provided with this paper. Source data includes raw data and statistical analysis data. This study makes use of the publicly available databases “CeNGEN (https://www.cengen.org)”, which is gene expression profiles of every neuron in C. elegans”

Research involving human participants, their data, or biological material

Policy information about studies with human participants or human data. See also policy information about sex, gender (identity/presentation), and sexual orientation and race, ethnicity and racism.

Reporting on sex and gender	This study is not "Research involving human participants, their data, or biological material".
Reporting on race, ethnicity, or other socially relevant groupings	This study is not "Research involving human participants, their data, or biological material".
Population characteristics	This study is not "Research involving human participants, their data, or biological material".
Recruitment	This study is not "Research involving human participants, their data, or biological material".
Ethics oversight	This study is not "Research involving human participants, their data, or biological material".

Note that full information on the approval of the study protocol must also be provided in the manuscript.

Field-specific reporting

Please select the one below that is the best fit for your research. If you are not sure, read the appropriate sections before making your selection.

☒ Life sciences ☐ Behavioural & social sciences ☐ Ecological, evolutionary & environmental sciences

For a reference copy of the document with all sections, see nature.com/documents/nr-reporting-summary-flat.pdf

Life sciences study design

All studies must disclose on these points even when the disclosure is negative.

Sample size	We determined sample size based on previous reports containing similar assays (Takeishi et al., Neuron 90, 235-244, 2016; Motomura et al., Proc Natl Acad Sci U S A 119, 2022). The sample sizes in dataset for cold tolerance assay, temperature acclimatization assay, and Ca2+ imaging in C. elegans shown in Fig. 1–3, Supplementary Fig. 2, 3 determined based on previous experiments using the same experimental procedures: at least 9 independent experiments were performed in these experiments. For Ca2+ imaging in Drosophila S2R+ cells in Fig. 4, Supplementary Fig. 4b, c, two to five independent trials were repeated three or more times on separate days. The number of biological replicates is described in the figure legend.<br>The sample size of this paper is sufficient, because we tested enough number of samples and enough number of assays, based on many previous reports containing similar assays.
Data exclusions	No data were excluded from the analyses.
Replication	All specific numbers regarding the frequency of repeat and independent experiments are given in the text. We have verified the reproducibility of all experimental groups.
Randomization	Allocations of all samples in this paper was random. We randomly picked the C. elegans animals from cultivation plate. We randomly selected S2R+ cells expressing a marker in the calcium-imaging experiments.
Blinding	We were blinded to group allocation during data collection and analysis.

Reporting for specific materials, systems and methods

We require information from authors about some types of materials, experimental systems and methods used in many studies. Here, indicate whether each material, system or method listed is relevant to your study. If you are not sure if a list item applies to your research, read the appropriate section before selecting a response.

Materials & experimental systems

n/a	Involved in the study
☒	☐ Antibodies
☐	☒ Eukaryotic cell lines
☒	☐ Palaeontology and archaeology
☐	☒ Animals and other organisms
☒	☐ Clinical data
☒	☐ Dual use research of concern
☒	☐ Plants

Methods

n/a	Involved in the study
☒	☐ ChIP-seq
☒	☐ Flow cytometry
☒	☐ MRI-based neuroimaging

Eukaryotic cell lines

Policy information about cell lines and Sex and Gender in Research

Cell line source(s)	We purchased Drosophila S2R+ cell line from Drosophila Genomics Resource Center (DGRC Stock 150 ; https://dgrc.bio.indiana.edu//stock/150 ; RRID:CVCL_Z831).
Authentication	The transcriptome of S2R+ is being mapped by the modENCODE project.
Mycoplasma contamination	The S2R+ cell line was not tested for mycoplasma contamination.
Commonly misidentified lines<br>(See ICLAC register)	We have described the source of the S2R+ cells in "Methods" section. The S2R+ cell is not on the list of known misidentified cell lines maintained by the International Cell Line Certification Committee (https://iclac.org/databases/cross-contaminations/). In addition, there have been no reports of cross-contamination for S2R+ cell.

Animals and other research organisms

Policy information about studies involving animals; ARRIVE guidelines recommended for reporting animal research, and Sex and Gender in Research

Laboratory animals	We used C. elegans strains originally stored in C. elegans stock center, CGC or NBRP. We listed all the original strains as bello. All animals used to collect data in the figures were adult. Well-fed adult animals having eggs were placed on a 35-mm dish containing NGM (nematode growth medium) for egg-laying, and the progeny were cultured for 120–150 h at 15°C, or 85–90 h at 20°C.<br><br>N2<br>eri-1(mg366); lin-15B(n744)<br>tax-4(p678)<br>osm-9(ky10)<br>ocr-2(ak47)<br>osm-9(ky10); ocr-2(ak47)<br>osm-9(ky10) ocr-2(ak47); ocr-1(ak46)<br>egl-30(ad806)<br>egl-30(ep271)<br>gcy-5(tm897)<br>glr-3(tm6403)<br>Ex[flp-6p::CeGCaMP8, gcy-5p::tagRFP]<br>glr-3(tm6403); Ex[flp-6p::CeGCaMP8, gcy-5p::tagRFP]<br>Ex[ges-1p::NLS::GFP, AIYp::GFP, pBluescript II SK+]<br>Ex[sre-1p::yc3.60, rol-6(gf)]<br>gpa-3(pk35)<br>goa-1(n1134)<br>goa-1(n1134); gpa-3(pk35)
Wild animals	We did not used field-collected C. elegans animals, in this study.
Reporting on sex	All C. elegans animals used to collect data in the figures were hermaphrodites. Male C. elegans were utilized for mating with hermaphrodites to generate double or multiple mutant strains
Field-collected samples	We did not used field-collected C. elegans animals, in this study.
Ethics oversight	Our university, Konan University, provided guidance on the study protocol.

Note that full information on the approval of the study protocol must also be provided in the manuscript.
